# Evaluation of nitrogen-delivery methods for stocker cattle grazing annual ryegrass

**DOI:** 10.1093/tas/txab048

**Published:** 2021-03-19

**Authors:** Phillip A Gunter, Mary K Mullenix, Lance C Burdette, Russell B Muntifering

**Affiliations:** 1 Department of Agriculture and Food Science, Western Kentucky University, Bowling Green, KY 42101, USA; 2 Department of Animal Sciences, Auburn University, Auburn, AL 36849, USA; 3 E.V. Smith Research Center, Shorter, AL 36075, USA

**Keywords:** annual ryegrass, cattle, clover, monensin, nitrogen, supplementation

## Abstract

A 2-yr grazing experiment was conducted to evaluate efficacy of nitrogen (N) fertilization, interseeded legumes, and protein supplementation for N delivery to stocker cattle grazing annual ryegrass (*Lolium multiflorum*). Each year, 90 steers (initial BW, 241 ± 13 kg) were assigned to the following N-delivery methods, with or without monensin fed in a free-choice mineral supplement as a 5 × 2 factorial arrangement of treatments: ryegrass fertilized with 112 kg N/ha (NFERT); ryegrass interseeded with crimson clover (CC, *Trifolium incarnatum*); ryegrass interseeded with arrowleaf clover (AC, *Trifolium vesiculosum*); ryegrass plus distillers dried grains with solubles (DDGS) supplemented at 0.65% BW daily; and ryegrass plus whole cottonseed (WCS) supplemented at 0.65% BW daily. Pastures within the interseeded-clover and protein-supplementation treatments were fertilized with 56 kg N/ha at time of establishment. Steers were weighed every 28 d, and forage mass (FM, kg DM/ha) was measured concurrently using the destructive harvest/disk meter double-sampling method. Each of 30 0.81-ha paddocks was stocked initially with 3 “tester” steers, and stocking density (steers/ha) was adjusted using “put-and-take steers” based on changes in FM and steer BW in order to maintain a uniform forage allowance (FA) of 1 kg DM/kg steer BW. Grazing was discontinued on May 11, 2016 in Yr 1 and May 10, 2017 in Yr 2 following 140 and 84 d of grazing, respectively. Data were analyzed as a completely randomized design with repeated measures for which pasture (*n* = 3) was the experimental unit. Ionophore inclusion did not affect (*P* > 0.10) any variable measured. Mean FM differed (*P* < 0.0001) between years and among N-delivery methods (*P* < 0.10), and mean FA differed (*P* = 0.005) among N-delivery methods. Steer ADG differed among N-delivery methods (*P* = 0.02) and between years (*P* < 0.001), whereas total gain/ha differed (*P* < 0.0008) among N-delivery methods, but not between years (*P* = 0.78). Stocking density differed among N-delivery methods (*P* = 0.02) and between years (*P* < 0.0001), and grazing-days/ha differed between years (*P* < 0.0001) and among N-delivery methods (*P* = 0.001). Results indicate that supplementation with a high-protein by-product feed for cattle grazing annual ryegrass maintained ADG, total gain/ha and grazing-days/ha compared with N-fertilized annual ryegrass, and increased ADG, total gain/ha, and grazing-days over interseeded legumes.

## INTRODUCTION

Input costs and high land prices are major challenges facing the beef cattle industry. To be economically viable, beef production systems must effectively exploit the capacity of the ruminant animal to consume and efficiently convert forage to liveweight gain. To realize this production strategy to its full potential, an abundant supply of high-quality forage must be continuously available. In the Southeast, this has traditionally been achieved by grazing small grains and other cool-season annual grasses (Utley et al., 1975). Annual ryegrass (*Lolium multiflorum* Lam.) is a cool-season annual bunchgrass (Hall, 1992) that can produce between 6,000 and 13,000 kg/ha of forage DM when fertilized adequately (Redfearn et al., 2002), and there are more than 1 million ha of ryegrass grown annually in the Southeast (Ball et al., 2007).

Nitrogen (N) fertilizer represents the single greatest variable-input cost of forage production for grazing by stocker cattle, and fertilizer costs rose steadily from the mid-1990s through the early 2010s, followed by a small decline in the late 2010s. Lower cost N-delivery alternatives such as interseeded legumes or supplementation with high-protein by-product feeds may provide a way to at least partially replace use of synthetic fertilizer in grazing systems. In the Southeast United States, use of regionally adapted legumes or locally available by-product feedstuffs high in CP may be economically advantageous to cattle producers. Crimson clover (*Trifolium incarnatum* L.) and arrowleaf clover (*Trifolium vesiculosum* Savi) are annual legumes with potential fit for integration into a cool-season annual-based stocker cattle grazing system (Ball et al., 2015).

Dried distillers grains with solubles (DDGS) and whole cottonseed (WCS) are readily available by-product feedstuffs in the Southeast United States that are used in drylot production systems (Mullenix and Rankins, 2014), but research evaluating supplementation of growing cattle with these high-CP feedstuffs on pasture is limited. Supplementation of stocker cattle with DDGS (2.3 kg∙animal^−1^∙d^−1^) increased ADG by 0.25 kg/d and total gain by 101 kg/ha over fertilized smooth bromegrass (Greenquist et al., 2009). Poore et al. (2006) observed an increase in ADG of 0.16 kg/d for heifers supplemented at 0.33% BW with WCS compared with unsupplemented heifers grazing stockpiled tall fescue. However, to date, no research has reported stocker cattle performance from use of these feedstuffs in cool-season annual forage systems. Incorporation of CP supplementation strategies along with other stacked management practices such as feeding monensin may potentially enhance grazing stocker cattle performance (Horn et al., 1981; Bretschneider et al., 2008).

The objective of this study was to evaluate the efficacy of N fertilization, interseeded legumes, and supplementation with high-protein by-products, with or without monensin, for N delivery to stocker cattle production from annual ryegrass.

## MATERIALS AND METHODS

All experimental procedures were implemented according to a protocol approved by the Auburn University Animal Care and Use Committee (PRN 2014–2438).

### Treatment Structure

Treatments were randomly assigned to 30 0.81-ha pastures in Yr 1 of a 2-yr study with the restriction that the same treatment could not be applied to adjacent pastures. Treatments in Yr 2 were maintained on the same pastures to which they had been assigned in Yr 1. Treatment structure was a completely randomized 5 × 2 factorial with 5 N-delivery methods, with or without monensin (Elanco, Greenfield, IN) provided in a custom-formulated compressed mineral block (Ridley Block Operations, Mankato, MN). Nitrogen-delivery methods included: annual ryegrass fertilized with 112 kg N/ha in a split-application (NFERT), annual ryegrass interseeded with crimson clover and fertilized with 56 kg N/ha at time of establishment (CC), annual ryegrass interseeded with arrowleaf clover and fertilized with 56 kg N/ha at time of establishment (AC), annual ryegrass fertilized with 56 kg N/ha and cattle supplemented with distillers dried grains with solubles at the rate of 0.65% BW (as-fed) daily (DDGS), and annual ryegrass fertilized at 56 kg N/ha and cattle supplemented with whole cottonseed at the rate of 0.65% BW (as-fed) daily (WCS). Supplement amounts (kg∙animal^−1^∙d^−1^) were adjusted every 28 d after cattle were weighed. Interseeding and supplementation rates were calculated to approximate the additional 56 kg N/ha that the NFERT treatment received in the second of the split application.

### Pasture Establishment

A 2-yr winter grazing trial was conducted at the E.V. Smith Research Center located in Milstead, AL (32.443°N lat., 85.897°W long.). Soil characteristics at initiation of the study were: 6.1 soil pH, 37 kg P/ha, 202 kg K/ha, 686 kg Mg/ha, and 2,118 kg Ca/ha. Thirty 0.81-ha paddocks that consisted of a fine sandy loam were used. Paddocks had previously been planted to annual ryegrass for the preceding 5 yr, and prior to that with warm-season grasses including bermudagrass (*Cynodon dactylon*), bahiagrass (*Paspalum notatum*), and dallisgrass (*Paspalum dilatatum*) for summer grazing.

Each year in early October prior to planting, pastures were fertilized with 329 kg 17-17-17 fertilizer to provide 56 kg N/ha. Pastures were planted on October 16, 2015 and December 23, 2016 in Yr 1 and 2, respectively; planting was delayed in Yr 2 compared with Yr 1 because of exceptionally dry soil conditions resulting from below-average precipitation and persistent drought conditions earlier in the fall. Each year, pastures assigned to NFERT, DDGS, and WCS were seeded at a rate of 34 kg/ha of “Marshall” annual ryegrass, and the interseeded clover pastures were seeded at a rate of 17 kg/ha of “Marshall” annual ryegrass and 34 kg/ha of “Dixie” crimson clover or 9 kg/ha of “Blackhawk” arrowleaf clover (Wax Company LLC, Amory, MS) to a depth of 0.6 cm into a prepared seedbed. Pastures assigned to the NFERT N-delivery method received an additional 56 kg N/ha as liquid N (28% N solution of ammonium nitrate and ammonium thiosulfate that provided 5% S) on February 23, 2016 and March 20, 2017.

### Animal and Pasture Management

Pastures were initially stocked with 90 crossbred “tester” steers (3 steers/pasture) of no more than ⅛ *Bos indicus* influence with an initial BW of 225 ± 10, 256 ± 15 in Yr 1 and 2, respectively. Cattle were procured through open-bid contract with a stocker producer in Reform, AL and were delivered in late December of each year. Upon delivery, calves were quarantined for 30 d on dormant mixed-grass paddocks and fed corn silage and grass hay at a maintenance level of intake in Yr 1. Due to delayed planting and turnout from drought in the late fall and early winter of Yr 2, calves were placed on dormant mixed-grass paddocks and fed a 50:50 blend of corn gluten feed and soybean hulls for a targeted gain of 0.25 kg/d. Prior to study initiation, calves were stratified by BW, randomly assigned to pastures and ear-tagged for identification. Calves were implanted with Ralgro (Merck Animal Health, Millsboro, DE) in Yr 1 and 2. Throughout the study, calves had access to clean water and a compressed mineral block with a targeted intake of 57–113 g∙animal^−1^∙d^−1^ that contained: 4.70–5.70% Ca, 4.0% P, 16.90–19.90% NaCl, 0.20% Mg, 1.50% K, 10 ppm Co, 1,000 ppm Cu, 140 ppm I, 3,950 ppm Mn, 13.3 ppm Se, 4,000 ppm Zn, 45,400 IU/kg Vit. A, 11,350 IU/kg Vit. D-3, and 11.35 IU/kg Vitamin E. Half of the blocks were nonmedicated and provided to the control group, and the other half contained monensin at 1,620 g/ton to provide 50–200 mg∙animal^−1^∙d^−1^. Supplement was provided once daily at approximately 0800 hours. Calves were weighed every 28 d following feed restriction for 24 h in order to derive shrunk weights. Weights were used to adjust supplement amounts for the succeeding 28-d period, and to adjust stocking densities in order to maintain a uniform forage allowance (FA) across all treatments of 1 kg forage DM/kg steer BW using “put-and-take” steers. Cattle were weighed and turned out onto pastures for grazing on December 14, 2015 (Yr 1) and February 15, 2017 (Yr 2). Mean initial forage mass (FM) was 1,068 and 539 kg DM/ha, and mean initial FA was 1.04 and 1.03 kg forage DM/kg BW in Yr 1 and 2, respectively. Grazing was terminated on May 11, 2016 (140 d) and May 10, 2017 (84 d) when forage quantity and quality could no longer maintain an ADG of 0.68 kg/d.

Forage mass was determined every 28 d when cattle were weighed using the double-sampling method described by Frame (1981). Twenty-five forage heights were recorded from each 0.81-ha pasture using a 0.25-m^2^ disk meter. Seventy-two calibration samples were taken from the 18 pastures assigned to the NFERT, CC, and AC treatments by recording forage heights and clipping forage to a stubble height of approximately 5 cm. Samples were placed in individual plastic bags and placed in a cooler for transport to the Auburn University Ruminant Nutrition Laboratory where they were transferred to individual paper bags and dried at 60°C to a constant weight. After drying, sample weights were plotted against their respective height values, and the resultant prediction equations were used to determine the forage DM mass for each pasture and to adjust stocking densities.

### Economic Evaluation

An economic evaluation of N-delivery methods was conducted to compare the N-fertilized pasture system with the interseeded-clover and protein byproduct-supplemented pastures on an input cost/ha and cost of gain basis. Economic values included variable-input costs of N fertilizer, labor, seed, supplements, and machinery. The hourly cost of equipment ($25.00) used during the experiment was determined previously by Prevatt et al. (2008) and multiplied by the hour of actual use time as recorded for each system. Diesel costs used were determined from the average retail cost of diesel during the 2 yr of the experiment. Labor costs were the number of hours of labor per system multiplied by $9.00/h. The price of 17-17-17, DDGS, and WCS were $415, $110, and $205/ton, respectively. Fertilizer and supplement costs were determined from the Alabama Weekly Feedstuff/Production Cost Report.

### Temperature and Precipitation

**Figure 1.**
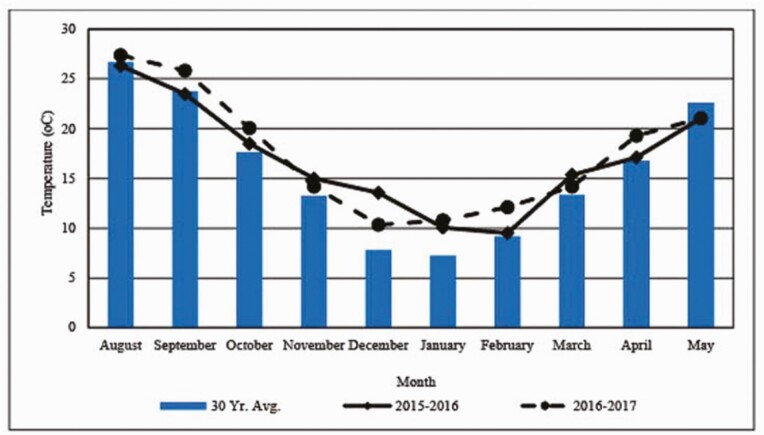
Monthly and 30-yr average temperatures from August to May by year at E.V. Smith Research Center, Milstead, AL.

**Figure 2.**
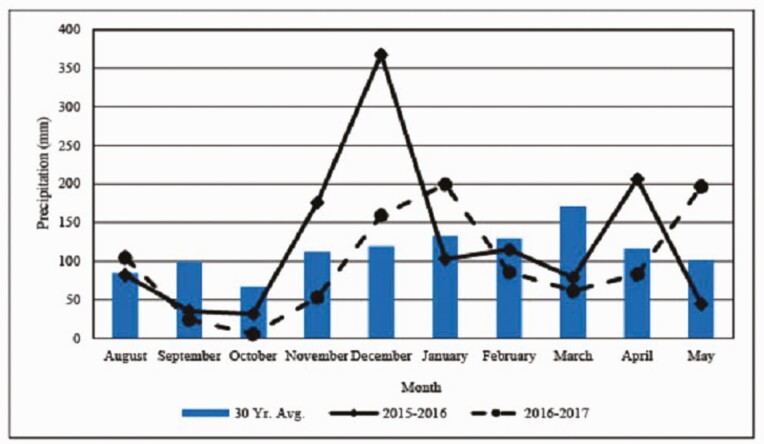
Monthly and 30-yr average monthly precipitation totals from August to May by year at E.V. Smith Research Center, Milstead, AL.

Monthly mean and 30-yr average monthly temperatures from August to May of each year at the research station are presented in Figure 1, and monthly and 30-yr average monthly precipitation totals from August to May of each year at the research station are presented in Figure 2. In Yr 1, monthly mean temperatures approximated 30-yr averages. Precipitation was less than average preceding and at time of planting in September and October, respectively, but greater than average rainfall in November and December allowed for very acceptable forage production with a start date for grazing of December 14, 2015. Adequate rainfall throughout the remainder of the winter and spring allowed for forage production that supported a typical 140-d grazing season. As in Yr 1, mean monthly temperatures in Yr 2 were very similar to 30-yr averages. However, precipitation was extremely low in September, October and November, which delayed planting until December 23, 2016. During and after December, rainfall was sufficient to support high forage mass production that enabled cattle to be turned out on February 15, 2017 for an 84-d grazing season.

### Statistical Analysis

Data were analyzed using PROC MIXED of SAS 9.4 (SAS Inst. Inc., Cary, NC.) for a 5 × 2 factorial design consisting of 5 N-delivery methods and with or without monensin. Data for all steers (i.e., “tester” and “put-and-take”) were used to determine stocking density, FA, and grazing d/ha. Total gain/ha was calculated for each pasture by multiplying ADG of “tester” steers by grazing-d/ha for both “tester” and “put-and-take” steers (Beck et al., 2011). Dependent variables evaluated included ADG, total gain/ha, stocking density, grazing-d/ha, FA, and FM. Month was included as a repeated measure for FM. Main effects were N-delivery method, ionophore, and year. Because there were no significant two- or three-way interactions detected for any of the dependent variables evaluated, their sums of squares and associated df were apportioned to the model error term (residual) for significance testing. The PDIFF option of LSMEANS was used to separate means when protected by F-test at *α* = 0.10, trends were declared at ≥ 0.10 to ≤ 0.15.

## RESULTS AND DISCUSSION

### Forage Metrics

Forage nutritive value and botanical composition of forages are presented and discussed in detail in Gunter et al. (2019). Briefly, forage CP concentration averaged 16% (DM basis) and IVTD averaged 88% (DM basis) across both years and all treatments, and clover abundance for interseeded treatments averaged 15% contribution for crimson clover and 0.65% for arrowleaf clover across both years. Forage mass (kg DM/ha) was different among N-delivery methods *(P* = 0.02; Table 1) and between years (*P* < 0.0001; Table 2). Forage mass was greater (*P* = 0.03) for NFERT, CC, DDGS, and WCS than AC, and was greater (*P* < 0.0001) in Yr 2 than Yr 1. Drought conditions in September and October of Yr 2 delayed planting, but greater than average rainfall in December and January boosted forage production potential. Precipitation and temperature in Yr 1 generally followed the 30-yr average, with elevated rainfall in November and December. The difference in timing of excess rainfall between Yr 1 and 2 may account for the greater forage mass observed in Yr 2, even though the grazing season was shortened due to drought the previous fall. Forage mass values of 2,061 kg DM/ha (Hafley, 1996) and 1,493 kg DM/ha (Mullenix et al., 2014) have been reported for continuously grazed annual ryegrass in multi-year grazing trials. These values are greater than those reported herein, which may be due in part to use of more conservative stocking rates in those studies as well as marked differences in weather conditions.

**Table 1.** N-delivery methods effect on cattle performance, grazing characteristics, and forage parameters

	N-delivery method^*^					
Item	NFERT	CC	AC	DDGS	WCS	SEM
ADG, kg/d	1.34^ab^	1.30^b^	1.25^b^	1.46^a^	1.43^a^	0.069
Total gain, kg/ha	528^a^	413^b^	389^b^	535^a^	515^a^	29.4
Stocking density, steers/ha	4.1^a^	3.5^b^	3.3^b^	3.7^b^	3.7^b^	0.15
Steer grazing-days/ha	395^a^	323^b^	311^b^	369^a^	369^a^	15.4
Forage mass, kg DM/ha	1,147^a^	1,063^a^	900^b^	1,096^a^	1,101^a^	58.2
Forage allowance, kg DM/kg BW	1.03^ab^	0.92^c^	0.99^bc^	1.05^ab^	1.07^a^	0.029

^a-b^Within a row, means without a common superscript differ (*P* < 0.10).

*NFERT, annual ryegrass fertilized with 112 kg N/ha in split application; CC, annual ryegrass fertilized with 56 kg N/ha and interseeded with crimson clover; AC, annual ryegrass fertilized with 56 kg N/ha and interseeded with arrowleaf clover; DDGS, annual ryegrass fertilized with 56 kg N/ha and cattle supplemented with dried distillers grains plus solubles at 0.65% BW/d; WCS, annual ryegrass fertilized with 56 kg N/ha and cattle supplemented with whole cottonseed at 0.65% BW/d.

**Table 2.** Year effect on cattle performance, grazing characteristics, and forage parameters

	Year^*^		
Item	1	2	SEM
ADG, kg/d	1.19^b^	1.52^a^	0.031
Total gain, kg/ha	480	472	18.6
Stocking density, steers/ha	2.9^b^	4.4^a^	0.09
Steer grazing-days/ha	395^a^	311^b^	9.7
Forage mass, kg/ha	913^b^	1,209^a^	36.6
Forage allowance, kg DM/kg BW	1.04	0.99	0.019

^a-b^Within a row, means without a common superscript differ (*P* < 0.10).

^*^Year 1, 2015–2016 grazing season; Year 2, 2016–2017 grazing season.

Forage allowance (kg DM/kg steer BW; Table 1) was affected (*P* = 0.0005) by N-delivery method such that FA was greatest for WCS that did not differ from DDGS or NFERT, NFERT was not different than AC, and AC was not different than CC, which was least. However, these differences were very small and not likely to have contributed to differences in cattle performance. Forage allowance tended (*P* = 0.13) to be slightly greater in Yr 1 than Yr 2. These values may be compared with those reported by Rouquette et al. (2018) from an 11-yr grazing experiment with bermudagrass pastures that were overseeded with annual ryegrass or arrowleaf clover to extend the warm-season grazing period. In their study, the relationship between calf ADG and ryegrass DM mass was optimized at a FA of approximately 1.3 and 1.5 kg DM/kg BW for arrowleaf clover and ryegrass, respectively. The relationship between ADG and FA has been reported to be more or less linear up to a FA of 3 kg DM/kg BW (McCartor and Rouquette, 1977). However, a nonlinear regression model indicated a FA of 1.8 was necessary to maintain an ADG of 0.9 kg (Beck et al., 2013). More recent reports indicate greater ADG at lesser FA than those reported by Beck et al. (2013). Mullenix et al. (2014) reported steer ADG of 1.2 kg/d with FA of 1.36 for annual ryegrass and small grain pastures. Similarly, Marchant et al. (2018) reported ADG of 1.44 kg/d at a FA of 0.89 for mixed annual ryegrass and small grain pastures. Weissend (2015) observed a mean ADG of 1.06 kg/d with a FA of 0.52 for annual ryegrass. Redmon et al. (1995) reported that ADG was not negatively impacted until a FA of 0.21 had been realized for small grain pastures.

### Cattle Performance

No differences (*P* > 0.10) were detected for any animal performance variable due to ionophore inclusion. The lack of response to monensin may have been due to the exceptionally high quality of the forage as noted above, and to management of stocking densities in order to maintain an adequate target FA of 1 kg DM/kg BW.

Average daily gain (kg/d) was greatest (*P* = 0.02; Table 1) for DDGS and WCS that were not different from NFERT, and was least for CC and AC that were not different from NFERT. Also, ADG was greater (*P* < 0.0001; Table 2) in Yr 2 than Yr 1. Bagley et al. (1988) evaluated steer performance from annual ryegrass fertilized at 34 kg N/ha, rye–annual ryegrass–arrowleaf clover, rye–annual ryegrass–ladino clover, and annual ryegrass–arrowleaf clover over 4 yr. They reported ADG of 1.00 kg/d for steers grazing annual ryegrass–arrowleaf clover and 0.93 kg/d for N-fertilized annual ryegrass. Mooso et al. (1990) reported an ADG of 0.97 kg/d for stocker cattle grazing annual ryegrass–white clover–crimson clover pastures. Mullenix et al. (2014) and Marchant et al. (2018) reported mean ADG of 1.37 and 1.44 kg/d for cattle grazing monocultures of triticale, wheat and annual ryegrass, or mixtures of these forages, respectively, that are similar to values reported in the current study. Weissend (2015) reported a mean ADG of 1.12 kg/d for cattle fed energy supplements when grazing annual ryegrass, and an ADG of 0.91 kg/d for unsupplemented cattle. Griffin et al. (2012) reported a linear increase in ADG as level of DDGS supplementation was increased from 0% to 0.6% to 1.2% BW (0.89, 1.03, 1.19 kg/d, respectively) for cattle grazing subirrigated Sandhills meadow. Greenquist et al. (2009) reported similar daily gains (0.92 kg/d) for cattle grazing smooth bromegrass and receiving DDGS at 0.6% BW/daily. Poore et al. (2006) supplemented heifers grazing tall fescue with whole cottonseed and reported an ADG of 0.51 kg/d, which is less than in the current study and due most likely to differences between forage species.

Total gain (kg/ha) was not different between year (*P* = 0.78; Table 2), but was greater (*P* = 0.0008; Table 1) for NFERT, DDGS, and WCS than CC and AC. Mooso et al. (1990) reported total gains of 494 and 532 kg/ha for steers grazing annual ryegrass–white clover and annual ryegrass–white clover–crimson clover pastures, respectively, which are greater than values in the current study and reflect longer grazing seasons. Hoveland et al. (1991) reported total gain of 575 kg/ha from annual ryegrass–crimson clover that they attributed to high stocking density and ADG. Hoveland et al. (1978) had previously reported total gains of 628 kg/ha from rye–arrowleaf clover–crimson clover, 473 kg/ha from ryegrass, and 460 kg/ha from arrowleaf clover–crimson clover overseeded into dormant bermudagrass. Marchant et al. (2018) reported a mean total gain of 541 kg/ha for steers grazing mixtures of wheat, triticale, and annual ryegrass. Greenquist et al. (2009) reported total gains of 404 kg/ha for DDGS-supplemented cattle grazing smooth bromegrass. Weissend (2015) reported total gains of 591 kg/ha for steers grazing annual ryegrass and fed energy supplements.

**Figure 3.**
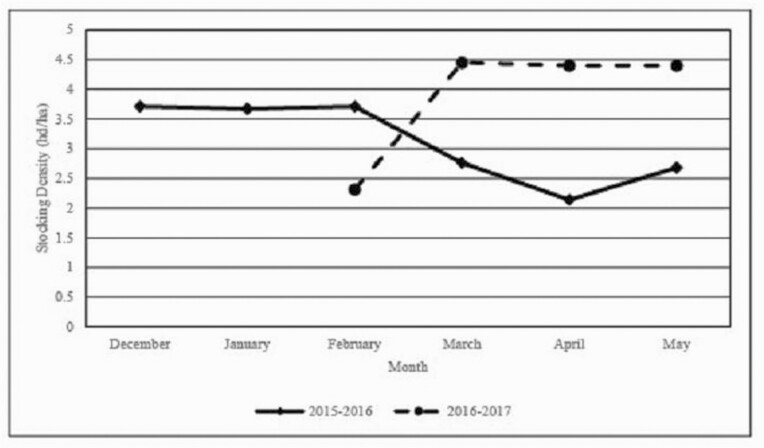
Monthly stocking density (steers/ha) changes from December to May by year.

Stocking density (steers/ha) was greater (*P* = 0.01; Table 1) for NFERT than CC, AC, DDGS, and WCS, and was greater (*P* < 0.0001; Table 2) in Yr 2 than Yr 1. Stocking densities were adjusted monthly in the current study based upon available forage DM. Forage mass in Yr 2 was plentiful due to favorable growing conditions of mild temperatures and greater-than-average rainfall in December and January when annual ryegrass and clovers, particularly crimson clover, were emerging and initiating vegetative growth. Weather conditions in Yr 1 were also favorable to cool-season forage production; however, the greater-than-average rainfall in November and December occurred well before forages had initiated vegetative growth. As such, forage productivity was less than in Yr 2. Stocking densities required to maintain a target FA of 1 kg forage DM/kg BW within all treatments were greater in Yr 1 from December through February but then declined thereafter, whereas stocking density was less in February and then increased until termination of the grazing season in May of Yr 2 (Figure 3).

Petersen et al. (1965) reported a positive linear relationship between gain/ha and stocking density, and that gain per animal was inversely related to stocking density once the rate at which forage was consumed surpassed the rate of growth of forage available for grazing. Furthermore, under- or overstocking may result in injury to and unwanted changes in sward composition. Bagley et al. (1988) reported stocking densities ranging from 2.84 to 3.14 steers/ha for cattle grazing mixtures of rye, ryegrass, arrowleaf clover, and ladino clover. These densities are less than those in the current study and may reflect use of subjective visual appraisal of pasture for adjusting stocking density as opposed to the double-sampling method used in this study. Hoveland et al. (1991) reported stocking densities of 5.29 steers/ha for mixed rye, annual ryegrass, and crimson clover pastures. Stocking densities of 3.5 and 2.94 steers/ha were reported by Mullenix (2014) and Marchant et al. (2018), respectively, for cattle grazing monocultures or mixtures, respectively, of wheat, triticale, and ryegrass.

Steer grazing-days/ha were greater (*P* = 0.001; Table 1) for NFERT, DDGS, and WCS than CC and AC, and greater (*P* < 0.001; Table 2) in Yr 1 than Yr 2. Steer grazing-days/ha were impacted by time of planting and forage productivity. Forage was planted at the typical time (October) in Yr 1, whereas drought delayed planting in Yr 2 until December. Cattle were placed onto pastures in December in Yr 1 and February in Yr 2. This delay reduced the available grazing-days in Yr 2 because these cool-season forages typically are productive from December to May or June (Ball et al., 2015). Weather conditions also impacted forage DM production as described above, with FM being greater in Yr 2 than Yr 1, which in turn impacted grazing-days/ha due to changes in stocking densities that were implemented to maintain a uniform forage allowance across treatments. Islam et al. (2011) reported greater number of grazing-days/ha for rye–annual ryegrass pastures (448) than tall fescue pastures (385). Mean grazing-days/ha of 375 and 439 were reported by Marchant et al. (2018) and Mullenix et al. (2014) for cattle grazing mixtures or monocultures, respectively, of ryegrass, triticale, and wheat. Myer et al. (2008) reported 366 grazing-days/ha for annual ryegrass.

### Economic Evaluation of N-Delivery Methods

Economic evaluations of the interseeded-clover and supplemented N-delivery methods compared with the NFERT treatment were conducted (Table 3). Variables included cost of N fertilizer (17-17-17), seed, supplement, labor, planting, fuel, and machinery costs. Labor and fuel costs for feeding supplements were not included, as these were incurred concurrently with daily checking of cattle in all treatments. Inputs costs ($/ha) for CC, AC, DDGS, and WCS were 76%, 60%, 59%, and 59%, respectively, of the input cost for NFERT. Cost of gain ($/kg) prorated over all steers for CC, AC, DDGS, and WCS were 102%, 84%, 57%, and 60%, respectively, of the cost of gain from NFERT. The discrepancy between the input costs and cost of gain, particularly for CC, was due to less gain/ha realized for CC than the supplemented treatments. The AC delivery method also supported less gain/ha than the supplemented treatments; however, because input costs were less for AC than CC, there was not as great a difference between input costs and costs of gain. Decisions on allocation of financial resources is based upon enterprise-specific considerations, and sustained profitability is predicated upon the ability of stocker producers to purchase calves in the fall of the year when prices are typically lower due to large supply of calves from cow–calf operators who do not want to feed and care for the calves during the winter (Rankins Jr. and Prevatt, 2013). The system that has historically provided the best opportunity for profitability is acquisition of lightweight calves in the fall and increasing BW by 100–200 kg for sale in the spring (Prevatt et al., 2011). Based upon the 2 yr of the current study, fertilization at half of the agronomic rate for annual ryegrass and provision of high-protein by-product feeds realized the least cost of gain.

**Table 3.** Estimated input costs ($/ha) and cost of gain ($/kg) associated with N-delivery methods for stocker cattle grazing annual ryegrass

	N-delivery method^*^				
Item	NFERT	CC	AC	DDGS	WCS
Fertilizer, $/ha	316.32	157.92	157.92	157.92	157.92
Seed, $/ha	25.16	107.85	33.58	25.16	25.16
Supplement, $/ha	0.00	0.00	0.00	3.65	6.82
Labor, $/ha	9.34	7.41	7.41	7.41	7.41
Fuel, $/ha	94.48	62.98	62.98	62.98	62.98
Machine cost, $/ha	25.93	20.68	20.68	20.68	20.68
Total input costs, $/ha	471.23	356.84	282.57	277.80	280.97
Cost of gain, $/kg	1.08	1.10	0.91	0.62	0.65

^*^NFERT, annual ryegrass fertilized with 112 kg N/ha in split application; CC, annual ryegrass fertilized with 56 kg N/ha and interseeded with crimson clover; AC, annual ryegrass fertilized with 56 kg N/ha and interseeded with arrowleaf clover; DDGS, annual ryegrass fertilized with 56 kg N/ha and cattle supplemented with dried distillers grains with solubles at 0.65% BW/d; WCS, annual ryegrass fertilized with 56 kg N/ha and cattle supplemented with whole cottonseed at 0.65% BW/d.

## SUMMARY AND CONCLUSION

Weather conditions and N-delivery method greatly impacted forage production and length of grazing season in the current study. Forage allowance was successfully maintained at 1 kg forage DM/kg steer BW by periodically adjusting stocking density on the basis of available forage DM. Average daily gain averaged 1.36 kg across treatments, with DDGS and WCS having the greatest gains. Total gain/ha, stocking densities, and grazing-d/ha were greater for NFERT, DDGS, and WCS than CC and AC. Average daily gain, stocking density, and grazing-days/ha differed between years due to variations in climatic conditions and date of grazing initiation. Cost of gain was least for treatments receiving supplement and greatest for the interseeded crimson clover treatment due primarily to lower gain/ha from the latter. Results indicate that supplementation with a high-protein by-product feed for cattle grazing annual ryegrass maintained ADG, total gain/ha and grazing-days/ha compared with N-fertilized annual ryegrass, and increased ADG, total gain/ha and grazing-days/ha over interseeded legumes. Feeding high-protein by-products may be more economically viable based upon input costs; however cattle purchase and sale prices may affect net income, which was not evaluated in the current study.
